# How to assess the accuracy of volume conduction models? A validation study with stereotactic EEG data

**DOI:** 10.3389/fnhum.2024.1279183

**Published:** 2024-02-12

**Authors:** Maria Carla Piastra, Robert Oostenveld, Simon Homölle, Biao Han, Qi Chen, Thom Oostendorp

**Affiliations:** ^1^Clinical Neurophysiology, Faculty of Science and Technology, Technical Medical Centre, University of Twente, Enschede, Netherlands; ^2^Department of Cognitive Neuroscience, Donders Institute for Brain, Cognition and Behaviour, Radboud University Medical Center, Nijmegen, Netherlands; ^3^Donders Institute for Brain, Cognition and Behaviour, Radboud University, Nijmegen, Netherlands; ^4^NatMEG, Karolinska Institutet, Stockholm, Sweden; ^5^School of Psychology, South China Normal University, Guangzhou, China

**Keywords:** volume conduction, EEG, stereotactic EEG, empirical validation, finite element method, head model

## Abstract

**Introduction:**

Volume conduction models of the human head are used in various neuroscience fields, such as for source reconstruction in EEG and MEG, and for modeling the effects of brain stimulation. Numerous studies have quantified the accuracy and sensitivity of volume conduction models by analyzing the effects of the geometrical and electrical features of the head model, the sensor model, the source model, and the numerical method. Most studies are based on simulations as it is hard to obtain sufficiently detailed measurements to compare to models. The recording of stereotactic EEG during electric stimulation mapping provides an opportunity for such empirical validation.

**Methods:**

In the study presented here, we used the potential distribution of volume-conducted artifacts that are due to cortical stimulation to evaluate the accuracy of finite element method (FEM) volume conduction models. We adopted a widely used strategy for numerical comparison, i.e., we fixed the geometrical description of the head model and the mathematical method to perform simulations, and we gradually altered the head models, by increasing the level of detail of the conductivity profile. We compared the simulated potentials at different levels of refinement with the measured potentials in three epilepsy patients.

**Results:**

Our results show that increasing the level of detail of the volume conduction head model only marginally improves the accuracy of the simulated potentials when compared to *in-vivo* sEEG measurements. The mismatch between measured and simulated potentials is, throughout all patients and models, maximally 40 microvolts (i.e., 10% relative error) in 80% of the stimulation-recording combination pairs and it is modulated by the distance between recording and stimulating electrodes.

**Discussion:**

Our study suggests that commonly used strategies used to validate volume conduction models based solely on simulations might give an overly optimistic idea about volume conduction model accuracy. We recommend more empirical validations to be performed to identify those factors in volume conduction models that have the highest impact on the accuracy of simulated potentials. We share the dataset to allow researchers to further investigate the mismatch between measurements and FEM models and to contribute to improving volume conduction models.

## 1 Introduction

Volume conduction models of the head are widely used for source reconstruction of electro- (EEG) and magnetoencephalography (MEG) activity (Malmivuo and Plonsey, 1995; Nunez and Srinivasan, 2006; Hansen et al., 2010), and are used to understand and optimize the effects of electrical (Neuling et al., 2012; Rampersad et al., 2014) and magnetic brain stimulation (Janssen et al., 2013) applied intra- and extracranially with transcranial electrical, deep brain, and magnetic stimulation (tES, DBS, and TMS). Although there are numerous model studies that quantified the accuracy of numerical approximations of electric potentials (in the EEG case) and magnetic fields (in the MEG case) by comparing different simulated models, there are fewer studies that investigated differences between actual measurements in humans and simulated potentials and fields (Rush and Driscoll, 1968; Bangera et al., 2010; Huang et al., 2017).

Previous work shows that the accuracy of the potential resulting from volume conduction models relies on a number of factors, such as the geometrical representation of the model (Vorwerk et al., 2014), the conductivity of the different tissues (Oostendorp et al., 2000; Aydin et al., 2014), the representation of the sensors (Pursiainen et al., 2012; Vermaas et al., 2020a), the representation of the sources [e.g., dipoles (De Munck et al., 1988) or bipoles (Vermaas et al., 2020b)], and the method used to solve the mathematical problem [e.g., with analytical formulas (de Munck and Peters, 1993; Zhang, 1995; Mosher et al., 1999), boundary element methods (Fuchs et al., 2001; Oostenveld and Oostendorp, 2002; Akalin-Acar and Gençer, 2004; Kybic et al., 2005; Stenroos and Sarvas, 2012; Makarov et al., 2020), finite difference methods (Montes-Restrepo et al., 2013; Morales et al., 2018; Moridera et al., 2021), and finite element methods (Marin et al., 1998; Schimpf et al., 2002; Miinalainen et al., 2019)].

The geometrical, electrical, and numerical aspects of volume conduction models are inherently interlinked. For example, the BEM assumes the geometry to be comprised of nested compartments with homogenous and isotropic conductivity, resulting in a geometrical description of the boundaries between compartments by triangulated surface meshes, where most BEM implementations require the surfaces not to touch or intersect, and where triangles should have a desired aspect ratio (Sun et al., 2012). Another example is the assumption of white matter conductivity being anisotropic, which limits the choice of the numerical method to FEM or FDM. The specific link between geometrical and electrical volume conduction model aspects is exemplified by including high-resolution anatomical details in the model, such as CSF, the compact and spongiform bone parts of the skull, blood vessels, or the dura mater, which require the geometrical description to have a spatial resolution that is high enough to be able to assign the detailed conductivities (Engwer et al., 2017; Piastra et al., 2018).

A strategy often adopted in validation studies that involve computer simulations is to focus on one or two of these factors and keep the remaining aspects fixed. In Nüßing et al. (2016), for example, the geometry of the head model was kept constant and the mathematical method to solve the forward problem was varied. In Piastra et al. (2018), the numerical method and the source model were changed, whereas the geometry was kept constant. In Vorwerk et al. (2014), the geometrical description and the numerical method were kept constant, and the conductivity profile was varied. Here, we adopt the same strategy as, e.g., in Vorwerk et al. (2014), keeping the identical geometry and numerical method, and explore the effects of increasing the level of detail in the head model by including more compartments with different conductivities. Going beyond existing simulation studies, we empirically compared the simulated potentials to measured potentials.

An interesting opportunity to empirically validate the forward model accuracy is provided by stereotactic EEG (sEEG) measurements during electric stimulation mapping, a technique used in the pre-surgical evaluation of epilepsy patients. Electrical stimulation mapping is essential for epilepsy surgery planning (Ritaccio et al., 2018), where pharmaco-resistant epileptic patients that are considered for resective surgery are implanted with intracranial (sEEG or electrocorticography (ECoG) electrodes to guide surgical resections of epileptiform tissue while sparing the eloquent cortex. In particular, by detecting behavioral changes, electrical stimulation is used to identify the epileptogenic zone or to localize the eloquent cortex which is to be spared in the subsequent resection. Electrical stimulation can also be combined with simultaneous recording of brain activity, resulting in cortical stimulation evoked potentials (CSEPs) that allow studying the spread of the induced activity, similar to how transcranial magnetic stimulation (TMS) evoked potentials are studied with scalp EEG (Bonato et al., 2006; Conde et al., 2019).

In the current study, we challenged the commonly employed strategy to improve volume conduction models based on the comparison between one simulation to another simulation, by validating volume conduction models using empirical data recorded from sEEG during stimulation. Rather than looking at the (biological) neuronal propagation of the activity of the CSEPs, we used the (physical) spatial potential distribution of the passively volume-conducted stimulation artifact. We compared the measured potential to the simulated potential that was computed with state-of-the-art FEM models based on the individuals' anatomical CT and MRI data of three epileptic patients. We investigate how the mismatch between recorded and simulated potentials depends on the level of detail in the FEM model, i.e., tissue conductivity, and on the distance between stimulating and recording sites.

## 2 Materials and methods

### 2.1 Ethics statement

Participants were recruited at the Guangdong Sanjiu Brain Hospital. The placement of the depth electrodes and the cortical stimulation were based solely on the clinical needs for the treatment of the patients and were thus independent of the purpose of the present study. This study did not add any invasive procedure to the intracranial recordings. The MRI, CT, and sEEG were all approved by the Ethics Committee of the School of Psychology, South China Normal University (SCNU-PSY-2020-1-050), and the Ethics Committee of Guangdong Sanjiu Brain Hospital. All the participants gave their written informed consent prior to the experiments in accordance with the Declaration of Helsinki.

### 2.2 Participants and data acquisitions

This study used data recorded for pre-surgical evaluation in three patients suffering from refractory epilepsy. The three participants (referred to as s1, s2, and s3) were 18, 21, and 25 years old. The three patients had 11, 9, and 15 semi-rigid multi-lead electrode shafts implanted, respectively. The electrode shafts had a diameter of 0.8 mm and contained 10–16 contacts that were 2 mm wide and 1.5 mm apart, with a total of 146, 124, and 186 electrodes per participant.

Intracranial sEEG recordings were conducted using commercial video-intracranial monitoring systems (Nihon Kohden). The data were bandpass filtered from 0.1 to 300 Hz and sampled at 1,000 Hz, using a reference electrode located in the white matter. During the CSEP recording procedures, around 40 electric stimulations were induced per patient in 20, 38, and 26 pairs of neighboring electrodes, respectively, while the sEEG signal was recorded on all remaining contacts. The total recording time was 17 m 15 s, 29 m 39 s, and 19 m 35 s, respectively.

Prior to the sEEG electrode implantation, T1 weighted spoiled gradient-recalled (SPGR) MRIs were acquired with a 3T scanner (GE Discovery MR750). Post-implantation CT images were acquired with a Philips Brilliance 64 scanner. The MRI resolution was 1.0 × 1.0 × 1.0, 0.5 × 0.5 × 0.5, and 0.5 × 0.5 × 0.5 mm for participants 1, 2, and 3, respectively, and the field of view (FOV) was 256 × 256 × 172, 390 × 435 × 418, and 374 × 424 × 377 for participants 1, 2, and 3, respectively. The CT resolution was 0.5 × 0.5 × 0.5 mm for all participants, and the FOV was 512 × 512 × 368, 390 × 435 × 418, and 374 × 424 × 377 for participants 1, 2, and 3, respectively.

### 2.3 Processing the sEEG data

All signal analysis was performed using FieldTrip (Oostenveld et al., 2011). The sEEG data was high-pass filtered at 10 Hz, segmented around the stimulation moments, and baseline corrected. Noisy channels were excluded following different criteria: variance of the after-peak signal higher than 10 millivolts, electrode positioned in the skull or scalp, and electrode close to or involved in the stimulation. The sEEG data were subsequently re-referenced to a bipolar montage (Allen et al., 1992), and the average of the peaks occurring at the moment of the electric stimulations was extracted.

### 2.4 Processing the anatomical MRI and CT data

Each participant's pre-implantation T1-weighted MRI scan was coregistered with the post-implantation CT scan, using rigid affine transformations derived from FSL's FLIRT algorithm (Jenkinson et al., 2002). The positions of sEEG electrodes were manually identified on the CT scan using the procedure outlined in Stolk et al. (2018). Some electrode contacts were located outside the brain and therefore not used in further analysis.

The MRI and CT scans of each patient were used to construct three individualized head models for each participant: a simple three-compartment isotropic head model (3C), where scalp, skull, and brain are included, a four-compartment isotropic head model (4C), where the cerebrospinal fluid (CSF) is additionally distinguished, and a more detailed volume conductor head model with five isotropic compartments (5C), i.e., scalp, skull, CSF, gray matter, and white matter.

To facilitate the segmentation procedure, the pre-implantation T1-weighted MRI scan and post-implantation CT scan were resampled so that the voxels of the anatomical data are cubic with 1 mm resolution. Furthermore, the images were truncated at 36, 30, and 35 mm below the spinal cord opening of participants 1, 2, and 3, respectively, following the suggestions in Lanfer et al. (2012).

As the T1-weighted anatomical MRI provides poor contrast to delineate the skull from the surrounding tissue, we segmented the skull from the CT scan by thresholding the CT scan, keeping the biggest connected components (performed in MATLAB), manually deleting electrodes and CT artifacts, and, finally, applying a smooth erode-dilate filter [performed in Seg3D (CIBC, 2016)]. The resulting skull geometry was closed, apart from the spinal cord opening, and skull burr holes drilled during surgery were excluded in the model.

The scalp, gray, and white matter compartments were segmented from the T1-weighted MRI scan using the SPM12 (Penny et al., 2011) routine implemented in FieldTrip. Finally, a series of binary operations was performed in Seg3D to combine the volumetric masks deduced from the two segmentations. In particular, the CSF compartment was constructed by subtracting the dilated inner skull mask from the scalp, skull, and gray and white matter masks. The dilation of the inner skull compartment was necessary to remove artificial holes at both the outer and inner skull interfaces generated by merging the skull segmentation from the CT and the one from the MRI. Since no DTI scans were acquired, we excluded the anisotropic conductivity tensors in the model (Tuch et al., 2001; Aydin et al., 2017).

Once the masks were assembled, a 1 mm volumetric hexahedral mesh was created (with a nodeshift of 0.3), resulting in ~3.5 million nodes and 3.5 million hexahedrons. For the three-compartment (3C) and four-compartment (4C) head models, only the tissue labels (and hence conductivities) were modified, while the mesh remained the same as the one for the five-compartment (5C) head model. The specific features of the three models are gathered in Table 1.

**Table 1:** Conductivity values (in S/m) of the three isotropic head models created and used in this study, with five (5C), four (4C), and three compartments (3C).

**Tissue**	**5C (S/m)**	**4C (S/m)**	**3C (S/m)**	**References**
White matter	0.14	–	–	Ramon et al., 2003
Gray matter	0.33	–	–	Ramon et al., 2003
Brain	:	0.33	0.33	Ramon et al., 2003
CSF	1.79	1.79	–	Baumann et al., 1997
Skull	0.01	0.01	0.01	Dannhauer et al., 2011
Scalp	0.43	0.43	0.43	Ramon et al., 2003, Dannhauer et al., 2011

The column (:) indicates that the compartment has been split, e.g., the brain compartment divided between gray and white matter, while the dash (-) indicates that the relative compartment has been neglected in the head model.

### 2.5 FEM simulations

The FEM simulations computing the electric potential difference distributions were performed using the DUNEuro software (Schrader et al., 2021). As Pursiainen et al. (2012) and Vermaas et al. (2020a) show that the spatial extent and geometry of electrodes do not play a significant role, we modeled the sensors as point-sensors. The stimulating electrode pairs were modeled as point-dipoles located at the midpoints between the neighboring anode and cathode.

### 2.6 Validation analysis/strategy

**Figure 1:**
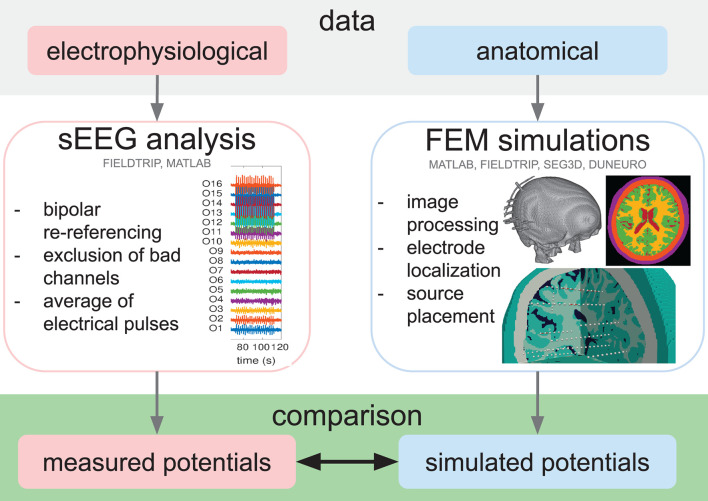
Schematic representation of the analysis pipeline. Following the collection of electrophysiological (sEEG) and anatomical (MRI and CT) data, the sEEG analysis was performed to obtain the measured potentials (red box), and the volume conduction simulations were set up, starting from the MRI and CT image processing, the electrode localization, and the source placement. Subsequently, the simulated potentials were computed (blue box) and the measured and simulated potentials were finally compared (green box). The software tools used in each step are indicated.

Figure 1 gives a schematic representation of the analysis pipeline. We computed the absolute difference between measured and simulated potentials for all combinations of the stimulation electrode pairs and all recording electrode pairs. Since the electrical stimulation pulse duration is only 0.3 ms and the sampling rate is 1,000 Hz (i.e., each sample represents 1 ms), the recording does not capture the full temporal detail of the rising and falling flank of the electrical stimulation and we were not able to retrieve the actual peak amplitude of the stimulation artifact in the data. We, therefore, determined a scaling factor that minimized the absolute error between simulated and measured potentials, i.e., 200, for all the participants and multiplied the simulated potentials by this factor.

**Figure 2:**
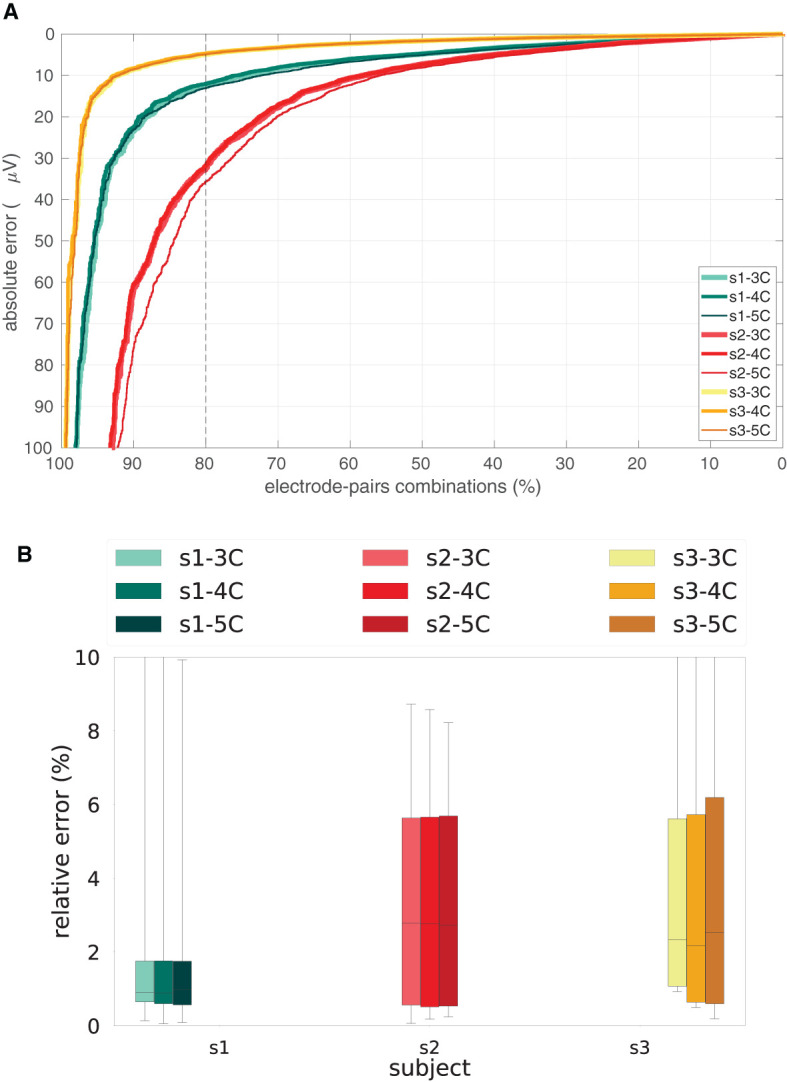
Absolute and relative errors per participant and head model. **(A)** Cumulative distributions in the percentage of the total number of stimulating-recording electrode pairs of the absolute difference between measured and simulated potentials in the three head models, i.e., 3C, 4C, and 5C, for the three participants, i.e., s1, s2, s3. The dashed vertical line represents 80% of the stimulation-recording combination pairs. **(B)** Boxplots of the relative difference between measured and simulated potentials in the three head models, i.e., 3C, 4C, and 5C, for the three participants, i.e., s1, s2, s3.

As we have around 30 stimulating electrode pairs which were each recorded with about 150 electrode pairs (channels), there are about 4,500 model simulations per participant and per volume conduction model to be compared to their corresponding measurements. We summarized the model errors over all stimulation sites and all recording sites by computing the cumulative distribution of the absolute differences between measured and simulated potentials. In addition, we normalized the absolute difference with the root mean square (RMS) over time and channels of the preprocessed signal, thus obtaining a relative difference between measured and simulated potentials. The RMS values are ~610, 332, and 96 μ*V*, for participants 1, 2, and 3, respectively. We investigated how the absolute and relative difference between measured and simulated potentials depends on the level of detail of the head model. In Figure 2, the absolute cumulative distribution curve and the relative error are visualized as boxplots.

**Figure 3:**
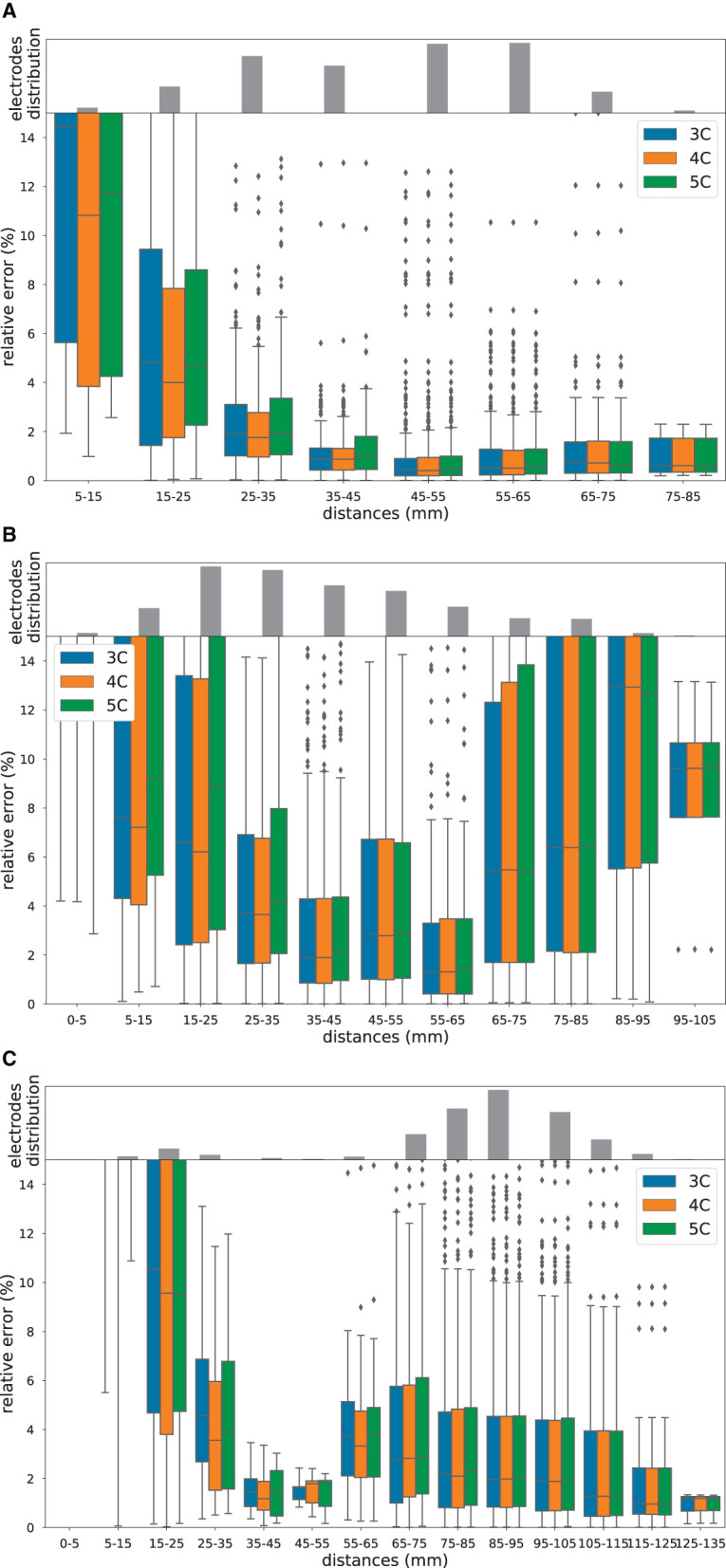
Relative errors and distances. Boxplot of the relative errors in percentage between measured and simulated potentials in 3C (in blue), 4C (in orange), and 5C (in green), for increasing distances between stimulating and recording electrode pairs, for participant 1 **(A)**, participant 2 **(B)** and participant 3 **(C)**. The gray bar plots on top of the boxplots are the histograms of the distances. Note the different scaling of the *y*-axis.

Furthermore, we studied how the relative error depends on the distance between stimulating and recording sites. To do so, we computed the distances between stimulation and recording sites, divided them into 5 mm bins, resulting in 9, 11, and 14 bins, respectively for the three participants, and visualized boxplots of the relative difference for each volume conduction model and for each participant (Figure 3).

## 3 Results

### 3.1 Difference between measured and simulated potentials for the three head models and participants

For each of the participants and for each of the volume conduction models we compared the measured potential to the simulated potentials for all combinations of stimulation and recording electrodes. Figure 2 shows the cumulative distribution (in the percentage of the total number of stimulating-recording electrode pairs) of the absolute error (top) and boxplots of the relative error (bottom) difference between the measured and simulated potentials in the three head models, i.e., 3C, 4C, and 5C, for the three participants, i.e., s1, s2, s3. From Figure 2 we can see that in 80% of the stimulation-recording electrode pair combinations, there is an absolute error of ≤ 5 (for s3), 15 (for s1), and 35 (for s2) μ*V* (Figure 2A), which corresponds to a relative error with median values of less than 1, 2.8 and 2.4%, respectively, (Figure 2B).

From Figure 2, we furthermore observe that the difference between the three head models (3C, 4C, 5C; line thickness) is considerably smaller than the difference between the three participants (s1, s2, s3; line color). In general, there is hardly any dependency of the error on the level of detail of the volume conduction head model used in the FEM simulation, since the cumulative distribution curves relative to 3C, 4C, and 5C are nearly overlapping for most participants.

### 3.2 Dependance of the simulation errors on the distance between stimulating and recording sites

In Figures 3A–C, we used a boxplot to visualize the relative error between measured and simulated potentials for different distances (in mm) between stimulating and recording electrode pairs, for the three head models and the three participants, i.e., s1, s2, and s3, respectively. Furthermore, we showed histograms of the distance distribution for each participant (gray bar plot on top of the boxplots).

From Figure 3, we observe that the relative error depends on the distance between the stimulating and recording electrode pairs. In particular, we can see that in all three participants, the error is very high for small distances and decreases for larger distances. For participant 2 and, to a smaller degree, also for participant 1, we can further notice that the relative error increases for distances larger than 65 mm. This effect is not present for participant 3 (Figure 3C).

All in all, without considering the closest stimulating-recording electrode combinations, i.e., the 0–5 mm bin, the medians of the relative errors are below 15% for all of the three participants. In particular, very low (2%) relative errors can be found for stimulating-recording electrode distances between 35 and 45 mm. Note that the distance histograms are very different among participants, which is not surprising considering that each epileptic patient has an individualized electrode implantation plan aligned with the clinical requirements and the suspected epileptogenic zones.

## 4 Discussion

In our study, we found that increasing the level of detail of the volume conduction head model only marginally improves the accuracy of the simulated potentials, when compared to *in-vivo* sEEG measurements of three epileptic patients undergoing electric stimulation mapping. In 80% of the stimulation-recording combination pairs, the relative error is around 10%; for stimulating-recording electrode distances between 35 and 45 mm, the relative error is 2%.

Many possible factors can explain why the difference among patients is much larger than the differences among models. One main difference lies in the different electrode implantation, the positioning and the number of electrodes are individualized and based on the collective assumptions of the epileptic network of the patient. This means that not only the density of sampling but also the within-tissue location of the electrodes are different. This can be partially seen, for example, in the three gray bar plots of Figure 3.

Another possible explanation for bigger differences among patients is the choice of fixed values for the conductivity profiles, whose modeling represents, in our opinion, the most delicate aspect to discuss. The standard approach used to include tissue conductivity in the volume conduction model relies on the MRI-based classification of the human head into a limited number of compartments; whereas FEM allows each volume element to have its own conductivity, we therefore only make use of a limited number of conductivity values. On top of this simplified compartmentalization, conductivity values are typically assigned independent of the individual characteristics of the participant. Moreover, template conductivity values are not consistent throughout the literature (McCann et al., 2019). Finally, there is no consensus on the method or technology that should be used to deduce or estimate such values (Ferrée et al., 2000; Verhoeven et al., 2015; Ranta et al., 2017; Acar and Makeig, 2022; Altakroury et al., 2022).

In literature, several direct and indirect volume conduction validation attempts have been carried out. In the pioneering work of Rush and Driscoll (1968), for example, simulated EEG potentials were tested both with a phantom, i.e., an electrolytic tank containing a human skull, and by comparison with *in-vivo* data from within the brain of a monkey (Burger and Van Milaan, 1943) and from the surface of a human head (Hayes, 1950). Given the technology available nowadays, it would be interesting to repeat such validation studies with, for example, 3D-printed phantoms that more accurately mimic the complexity of human head tissues (Avery et al., 2017; Zhang et al., 2017; Tsizin et al., 2018; Morales-Quezada et al., 2019; Kuratko et al., 2022).

Both in our study and in Bangera et al. (2010), it is found that increasing the level of detail of the volume conduction model does not improve the accuracy of the iEEG forward simulations within the skull compartment. Bangera et al. made use of stereo EEG recordings of four epileptic patients to assess which level of detail should be adopted in volume conduction modeling of the inner skull head. Similar to our work, they computed FEM solutions in a variety of head models with an increasing number of compartments included in both isotropic and anisotropic models. Differently from Bagera, where they restricted their claims to intracranial EEG forward solutions accuracy, the overall goal of our study is to have a critical eye on how the accuracy of forward simulations is assessed in literature, independent of whether intra or extracranial compartments are included in the model.

In a reciprocal sense (see, e.g., Vallaghé et al., 2008; Wagner et al., 2016), validation conducted for transcranial electrical stimulation (tES) simulations can be associated with and compared to forward solutions validations. In Opitz et al. (2016), for example, sEEG recordings during extracranial tES stimulations were acquired and analyzed in monkeys and humans. In their study, the focus is on assessing the intracranial amplitude of the potential induced by tES, and did not study the effect of variations in the volume conductor model, e.g. by varying the number of compartments. In Datta et al. (2013), the attempt on characterizing scalp voltages generated by tES to validate participant-specific FEM models of current flow for clinical dose is presented. They concluded that the FEM model accurately predicted the distinct voltage distributions and correlated the induced scalp voltages with current flow through the cortex, without directly validating the model prediction of brain current flow.

In recent work of Huang et al. (2017), the influence of different volume conduction head models is quantified in a tES/sEEG validation. Despite multiple refinements in the head model, Huang et al. (2017) did not find consistently that a more complex model improves the simulation performance across the ten participants analyzed in their work. Our findings are therefore also in line with their conclusions.

One indirect way to validate volume conduction models is to compare source reconstruction results with known intracranial sources, in terms of source location, magnitude, and orientation. In several studies (e.g., in Cohen et al., 1990; Murakami et al., 2016; Mikulan et al., 2020; Unnwongse et al., 2023), such validation has been performed without, nevertheless, studying the influence of the forward model accuracy on the source reconstruction results. In contrast, Leahy et al. (1998) performed such validation in a three-layered human phantom, concluding that the influence of using a realistic head model instead of a sphere for computing the forward solution was found to be minimal on the location mismatch. In addition, Gullmar et al. (2006) and Lau et al. (2016) made use of *in-vivo* measurements of a rabbit implanted with actual dipoles to study the influence of white matter anisotropic conductivity (Gullmar et al., 2006), and skull defects (Lau et al., 2016), on EEG (Gullmar et al., 2006), and MEG (Lau et al., 2016), source reconstruction. Gullmar et al. (2006) found a strong influence of the anisotropy on the magnitude in the forward as well as in the inverse solution and on the orientation of dipoles in the inverse solution. They concluded that source localization procedures in animals will improve by including white matter anisotropy. In Lau et al. (2016), the forward simulation of the MEG signals reproduced the experimentally observed characteristic magnitude and topography changes due to skull defects. They conclude that detailed finite element head models can improve the localization of brain function, specifically after surgery.

Despite our best efforts, there remains some model inaccuracy that are related to data limitations. For example, partial volume effects might lead to inaccurate brain structure quantification in MRIs or electrode identification in CTs.

Moreover, there are features of the sEEG signal we cannot fully take into account in our study due to limitations in recording hardware. Both the onset and the offset of the pulse are affected by capacitive filtering effects that last more than a millisecond. As a result, the pulse (which has a duration of 0.3 ms) ends well before the capacitive effect from the start has subsided. In addition, since the sample period (1 ms) is longer than the pulse duration, the recorded potential cannot be related directly to the stimulation strength. As already mentioned, we, therefore, adopted a scaling factor of 200 to fit the simulated potentials into the measured potentials which compensates for the uncertain, but fixed, relation between the stimulation strength and the average of the recorded potentials. Since the technical features of the stimulation and recording setup were the same for all participants, we believe it is appropriate to assume the same scaling factor for all participants. The scaling factor does not affect the main conclusion of this work, since we would notice a consistent global rescaling of the error curves and bars in Figure 2, but the relation between curves with different head models would be untouched. We are nevertheless planning to perform a similar study with higher sample rates and longer pulse durations.

As to model inaccuracies, we know that assuming point-like dipoles introduces modeling errors at small distances, see Figure 3, which can be reduced if monopole models are adopted instead. We recently developed a tool, i.e., FEMfuns (Vermaas et al., 2020b), that is able to model monopolar sources in volume conduction simulations, and we are planning to use it in the future. Nevertheless, this inaccuracy does not explain why more detailed head models do not lead to more accurate FEM solutions in our study. We believe that a U-shaped behavior with a subsequent descent slope might be present in all subjects, but only part of this shape is visible given the electrode configuration, i.e., distance. In all subjects, the errors are the highest for the smallest distances and decrease until reaching a minimum at around 35-65 mm. Subsequently, the errors are increasing again for higher distances, describing a U-shape. What is visible for subjects 2 and 3 (and not for subject 1) is that after around 95 mm for subject 2 and 75 mm for subject 3, the errors are decreasing with higher distances. While the U-shaped behavior of the errors in the vicinity of the sources (i.e., dipolar vs monopolar source model) and in the proximity of conductivity jumps (at higher distances) is clear, further analysis is required to understand the behavior of the errors for further higher distances.

Though they could only be important for intracranial recording simulations, CSF shunting effects in the electrodes' vicinity are not sufficiently captured by our volume conduction model. Similar to what is demonstrated in Vermaas et al. (2020a), more accurate features of the electrodes, such as volumetric extent, shape, and electrical properties, can be neglected since we are looking at distances higher than the dimension of the electrodes themselves. However, by not including the electrode structure, we are neglecting a possible CSF layer that is around the electrodes and relative shunting effects. During surgery, holes are drilled in the skull and the electrode shaft is inserted into the brain, allowing CSF to flow between the shaft and neighboring tissue.

There exist more sophisticated numerical methods to solve the quasi-static approximation of Maxwell's equations. Recently, for example, the discontinuous Galerkin FEM (Engwer et al., 2017; Piastra et al., 2018) was shown to alleviate modeling inaccuracies that occur in head geometries when using classical FE methods, e.g., so-called “skull leakage effects” for skull compartments with a thickness in the range of the mesh resolution (Engwer et al., 2017). Since we are focusing on the model accuracy in the inner skull compartment, our study does not fall in the scenario where the DG-FEM can be beneficial and therefore we do not expect these numerical solutions to substantially improve the accuracy in our study.

All in all, in the last decades, a lot of effort has been directed toward improving volume conduction models in terms of geometrical approximation (Vorwerk et al., 2014), source representation (Riera et al., 2012; Gratiy et al., 2013) and discretization (Haueisen et al., 1997; von Ellenrieder et al., 2006), and numerical accuracy (Engwer et al., 2017; Miinalainen et al., 2019), each individually showing incremental improvements. However, comparing the mismatch between measured and simulated potentials found in our study, the improvements in FEM models achieved in recent years that we were able to incorporate in our forward models appear relatively marginal and result in a limited accuracy compared to real data.

Considering our results, we feel that the commonly employed strategy to improve volume conduction models based on the comparison between one simulation to another simulation might not be the most efficient, we rather might want to reorient and channel more efforts toward actual measurements and empirical validations. We believe that empirical validations are more likely to reveal which aspects of the data, of the model assumptions and/or methodological details have the most impact to improve model potential distributions, for example, working with higher resolution imaging data and model geometries, better use of template anatomical models to deal with details that are too small to be imaged, and improved approaches for conductivity estimation such as Bayesian (Stahlhut et al., 2011) or deep learning techniques (Rashed et al., 2020).

Finally, we share the dataset of this study to allow researchers to shed new light on the reasons behind the high mismatch and to contribute to improving volume conduction models.

## 5 Conclusions

From our empirical comparison of FEM volume conduction model simulations with *in-vivo* measured sEEG potentials, we conclude that even with state-of-the-art model, increasing the level of detail of the volume conduction head model only marginally improves the accuracy of the simulated potentials when compared to the measurements. We argue that commonly employed methods for validating volume conduction models that rely solely on simulations should be supplemented with empirical validations based on actual data, as these will highlight the volume conduction model elements that have the greatest influence on the accuracy of simulated potentials.

## Data availability statement

The data and code that support the findings of this study are available from the Donders Repository (https://doi.org/10.34973/j0jh-9j28).

## Ethics statement

The studies involving humans were approved by Ethics Committee of the School of Psychology, South China Normal University (SCNU-PSY-2020-1-050), and the Ethics Committee of Guangdong Sanjiu Brain Hospital. The studies were conducted in accordance with the local legislation and institutional requirements. The participants provided their written informed consent to participate in this study.

## Author contributions

MCP: Conceptualization, Formal analysis, Investigation, Methodology, Project administration, Software, Visualization, Writing – original draft, Writing – review & editing, Validation. RO: Methodology, Software, Writing – review & editing, Conceptualization, Supervision. SH: Writing – review & editing, Investigation, Software. BH: Data curation, Writing – review & editing, Resources. QC: Writing – review & editing, Data curation, Funding acquisition. TO: Conceptualization, Funding acquisition, Methodology, Supervision, Writing – review & editing, Investigation.
